# Melatonin Treatment Delays Senescence and Maintains the Postharvest Quality of Baby Mustard (*Brassica juncea* var. *gemmifera*)

**DOI:** 10.3389/fpls.2021.817861

**Published:** 2022-01-28

**Authors:** Hongmei Di, Zhiqing Li, Yating Wang, Yi Zhang, Jinlin Bian, Jingyi Xu, Yangxia Zheng, Ronggao Gong, Huanxiu Li, Fen Zhang, Bo Sun

**Affiliations:** ^1^College of Horticulture, Sichuan Agricultural University, Chengdu, China; ^2^Institute of Pomology and Olericulture, Sichuan Agricultural University, Chengdu, China

**Keywords:** melatonin, baby mustard (*Brassica juncea* var. *gemmifera*), sensory, antioxidant, glucosinolate

## Abstract

The effect of melatonin treatment on the visual quality and content of health-promoting compounds in baby mustard (*Brassica juncea* var. *gemmifera*) at 20°C was investigated in this study. Application of 100 μmol L^–1^ melatonin was the most effective in prolonging the shelf life of baby mustard among all of the concentrations tested (1, 50, 100, and 200 μmol L^–1^). The 100 μmol L^–1^ melatonin treatment also delayed the increase in weight loss and the decrease in sensory parameter scores; retarded the decline of chlorophyll content; slowed the decline in antioxidant capacity by maintaining the content of carotenoids and ascorbic acid, as well as increasing the levels of total phenolics; and increased the content of individual and total glucosinolates in the lateral buds of baby mustard. These findings indicate that melatonin treatment is effective for maintaining the sensory and nutritional qualities of postharvest baby mustard.

## Introduction

Baby mustard (*Brassica juncea* var. *gemmifera*) is becoming increasingly popular among consumers for its sensory and nutritional properties. The lateral buds are tender with a sweet and fragrant flavor. Baby mustard contains numerous health-promoting compounds including ascorbic acid, carotenoids, phenolics, and glucosinolates (Sun et al., 2019). However, the lateral buds of baby mustard are susceptible to dehydration, browning, and the loss of health-promoting compounds during postharvest storage at room temperature (Sun et al., 2018, 2021). Several methods have been used to preserve the quality of postharvest baby mustard, such as long-term freezing treatment (Zhang et al., 2021), low temperature storage (Sun et al., 2020), and light exposure (Sun et al., 2021). Given the inconvenience and high costs of these methods, there is a need to develop improved techniques to extend the shelf life and preserve the postharvest quality of baby mustard during storage at room temperature.

Melatonin (*N*-acetyl-5-methoxytryptamine) is a derivative of tryptophan that occurs in all living organisms, including bacteria, fungi, plants, and mammals (Tang et al., 2020; Wu X. et al., 2021). This indoleamine is a pleiotropic molecule with a wide range of cellular and physiological functions in plants (Arnao and Hernández-Ruiz, 2019; Deng et al., 2021). In addition to its key role in plant growth and development, melatonin has been shown to be effective in promoting the postharvest quality of fruits and vegetables, including delaying senescence, controlling disease, and alleviating chilling injury (Zhang et al., 2020). A delay in browning and other forms of quality deterioration caused by melatonin have been observed in numerous horticultural plants, such as peaches (Cao et al., 2016; Gao et al., 2016), pears (Zhai et al., 2018; Zheng et al., 2019), cassavas (Ma et al., 2016), bamboo shoots (Li et al., 2019), broccoli florets (Miao et al., 2020; Wei et al., 2020; Wu C. H. et al., 2021), and Chinese flowering cabbages (Tan et al., 2019, 2021). For example, melatonin treatment has been reported to delay the senescence of Chinese flowering cabbages by suppressing chlorophyll degradation and ABF-mediated abscisic acid biosynthesis (Tan et al., 2019). Melatonin treatment has also been shown to maintain the chlorophyll content in broccoli florets (Miao et al., 2020; Wu C. H. et al., 2021). Melatonin is widely known to be a direct scavenger of reactive oxygen species (ROS). Melatonin treatment can also reduce the accumulation of ROS indirectly by enhancing the activity of the ROS-scavenging system including major antioxidant enzymes (SOD, CAT, APX, etc.) and other natural antioxidants (carotenoids, ascorbic acid, phenolics, etc.) (Gao et al., 2016). It can also increase the levels of beneficial substances, such as amino acids, sugars, and soluble solids and thus enhance the nutritional quality of apples (Onik et al., 2021) and plums (Bal, 2019) postharvest. Furthermore, it can maintain the content of glucosinolates and positively affect the glucoraphanin-sulforaphane system in broccoli florets (Miao et al., 2020; Wei et al., 2020). These findings indicate that melatonin can be used for the postharvest preservation of fruits and vegetables because of its multiple effects.

Although melatonin treatment has been shown to be useful for the postharvest preservation of various fruits and vegetables, there is little information on the effect of melatonin on postharvest baby mustard. The aim of the current study was to explore the effects of melatonin on the sensory quality, chlorophyll, carotenoids, ascorbic acid, total phenolics, antioxidant capacity, and glucosinolates in baby mustard during storage.

## Materials and Methods

### Plant Materials and Melatonin Treatments

Baby mustard (*B. juncea* var. *gemmifera*), harvested early in the morning, was obtained from a local farm in Chengdu City, China, and transported to the laboratory within 2 h under ambient temperature. In experiment I, 120 lateral buds were randomly divided into five batches in quadruplicate (six lateral buds per replicate) and were immersed in 0 (control), 1, 50, 100, and 200 μmol L^–1^ melatonin solutions for 5 min. The lateral buds were then removed from the solutions and air-dried. All the procedures were performed at room temperature. Afterward, each replicate was placed in a transparent polypropylene container and stored at 20°C with a relative humidity of 75% for 6 days. Samples were treated and stored in the dark (<0.01 μmol m^–2^ s^–1^) to prevent melatonin decomposition. Treatment with 100 μmol L^–1^ melatonin significantly extended the shelf life of baby mustard compared with other treatment groups and the control.

In experiment II, a total of 168 lateral buds were assigned to two groups (0 and 100 μmol L^–1^ melatonin). Similarly, there are four replicates in both groups, and each replicate contains six lateral buds. Samples were taken before melatonin treatment (time 0) and at 2-day intervals during storage for measurements. Among the six lateral buds in each replicate, three lateral buds were tested for analyses of sensory quality and weight loss; the remaining three are lyophilized and stored at −20°C for further analyses of phytochemicals and antioxidant capacity.

### Shelf Life and Sensory Quality Evaluation

Shelf life and sensory quality of the baby mustard lateral buds were assessed daily and on sampling day, respectively. The lateral buds were considered to have reached the end of their shelf life when they became soft, shrank, and exhibited browning (Sun et al., 2021). Sensory attributes were quantified on a scale from 5 (best) to 1 (worst). Color was rated using 5 = bright green without defects, 3 = lighter green with a few browning spots, and 1 = yellowish lateral buds with severe browning. Form was rated using 5 = the leaves on the upper of lateral buds are fresh and straight, 3 = shrinkage appears to the leaves of lateral buds, and 1 = the leaves shrink significantly. Odor was rated using 5 = no off-odors, 3 = slight but obvious off-odor, and 1 = strong off-odor. Texture was rated using 5 = very tight and firm, 3 = slightly soften but acceptable, and 1 = very soften. Acceptance was rated using 5 = excellent and having a freshly harvested appearance, 3 = average, and 1 = unmarketable.

### Weight Loss

Weight loss (%) was calculated by the formula (W_*x*_ - W_0_)/W_0_ × 100, where W_0_ is the weight at 0 day, and W_*x*_ is the weight at a certain day after storage (Sun et al., 2021).

### Chlorophyll and Carotenoid Content

The powder of lateral bud was ground and extracted with acetone, and the supernatant was filtered and analyzed by high-performance liquid chromatography (HPLC). Samples (10 μL) were separated using isopropanol and 80% acetonitrile–water at a flow rate of 0.5 mL min^–1^ (Sun et al., 2021).

### Ascorbic Acid Content

The sample powder was extracted with 1.0% oxalic acid, and then centrifuged. Each sample was filtered through a 0.45 μm cellulose acetate filter, and analyzed by HPLC. The amount of ascorbic acid was calculated from absorbance values at 243 nm (Sun et al., 2018).

### Total Phenolics Content

Total phenolics were extracted with 50% ethanol, and the supernatant was mixed with Folin-Ciocalteu reagent, after 3 min, saturated sodium carbonate was added. The absorbance was measured at 760 nm with the spectrophotometer (Sun et al., 2018).

### Ferric Reducing Antioxidant Power

The extracted samples were added to the ferric reducing antioxidant power (FRAP) working solution incubated at 37°C. The absorbance was then recorded at 593 nm using a spectrophotometer after the mixture had been incubated in at 37°C for 10 min, and then the value was calculated (Sun et al., 2018).

### 2,2-Azinobis (3-Ethyl-Benzothiazoline-6-Sulfonic Acid) (ABTS) Assay

An aliquot of 300 μL of each extracted sample was added to 3 mL of ABTS^+^ solution. The absorbance was measured spectrophotometrically at 734 nm after exactly 2 h, and then the value was calculated. The percentage inhibition was calculated according to the formula:% inhibition = [(A_*control*_ - A_*sample*_)/A_*control*_] × 100% (Sun et al., 2018).

### Glucosinolate Composition and Content

Freeze-dried samples (100 mg) were boiled in 5 mL water for 10 min to destroy the activity of myrosinases and prevent glucosinolates from hydrolysis. The supernatant was collected and applied to a DEAE-Sephadex A-25 column. The glucosinolates were converted into their desulpho analogues by treated with aryl sulphatase, and the desulphoglucosinolates were eluted, and then analyzed by HPLC. Glucosinolates were quantified by using *ortho*-Nitrophenyl β-D-galactopyranoside as the internal standard and considering the response factor of each glucosinolate (Sun et al., 2018).

### Data Analysis

Data were analyzed using one-way ANOVAs. A time-related trajectory analysis based on a principal component analysis map was used to visualize temporal changes in postharvest quality between different treatments (Sun et al., 2021).

## Results

### Shelf Life

**FIGURE 1:**
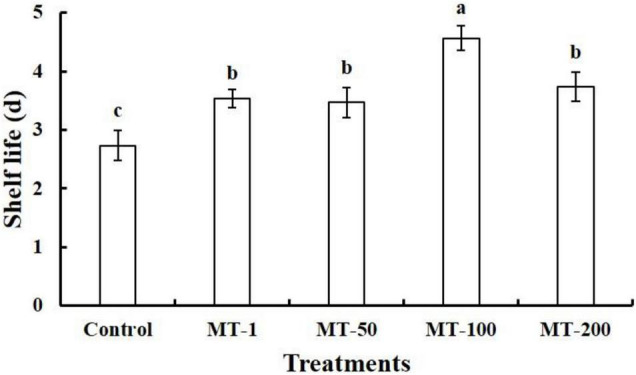
Shelf life of different concentrations of melatonin-treated lateral buds of baby mustard during storage at 20°C. Different letters in the figure indicate statistically significant differences among treatments (*P* < 0.05). MT-1, 1 μmol L^–1^ melatonin treatment; MT-50, 50 μmol L^–1^ melatonin treatment; MT-100, 100 μmol L^–1^ melatonin treatment; and MT-200, 200 μmol L^–1^ melatonin treatment.

In experiment I, all of the melatonin treatments significantly extended the shelf life of the lateral buds of baby mustard (Figure 1). The shelf life was the longest for the 100 μmol L^–1^ melatonin treatment (4.6 days), which was 1.7-fold greater than that of the control (2.7 days). No significant differences in the shelf life were observed between the other three melatonin treatments (average of 3.4 days). Thus, 100 μmol L^–1^ melatonin was used in subsequent experiments.

### Sensory Quality

**FIGURE 2:**
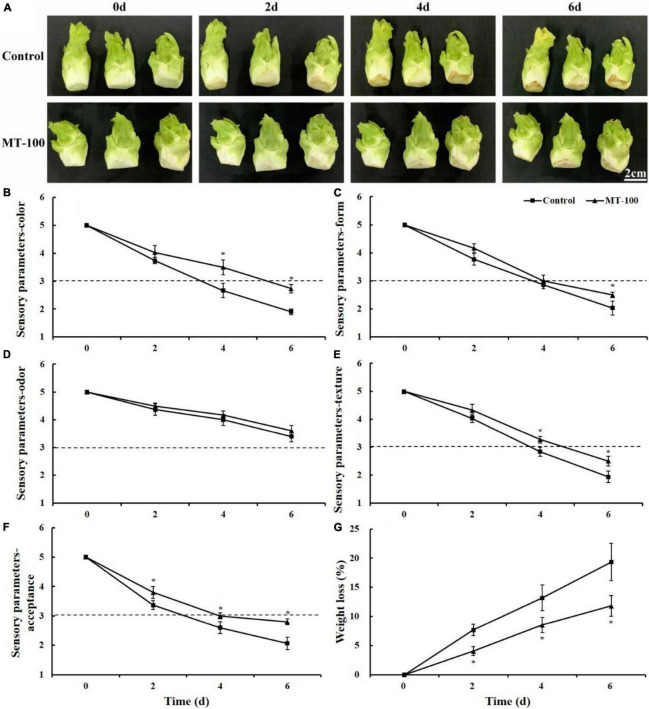
Sensory quality and weight loss of 100 μmol L^–1^ melatonin-treated lateral buds of baby mustard during storage at 20°C. **(A)** Lateral buds of baby mustard at each sampling time. **(B–F)** Sensory parameters including color, form, odor, texture, and acceptance of lateral buds. **(G)** weight loss. MT-100, 100 μmol L^–1^ melatonin treatment. Each value is presented as the mean ± standard error of four biological replicates. Asterisks (*) indicate the significant differences (*P* < 0.05) between control and melatonin-treated lateral buds during storage.

The treatment of 100 μmol L^–1^ melatonin notably suppressed the deterioration of the external aspect of the lateral buds. The control group showed obvious browning and yellowing at 4 and 6 days, respectively. In the melatonin-treated group, slight browning was observed at 6 days (Figure 2A).

At 4 days, the sensory parameter scores, with the exception of odor, were less than 3 in the control, which means that they were unsaleable (Figures 2B–F). The treatment with 100 μmol L^–1^ melatonin had significantly higher color, form, texture, and acceptance scores compared with the control at 6 days (Figures 2B,C,E,F). The color and acceptance scores of the melatonin-treated group at 6 days were still higher than those of the control at 4 days of storage (Figures 2B,F). No differences in the odor scores were observed between the control and melatonin treatment during the entire storage period and were 3.4 and 3.6 at 6 days, respectively (Figure 2D).

### Weight Loss

Weight loss increased regardless of whether the lateral buds were treated with melatonin during storage (Figure 2G). The weight loss observed during the storage period was significantly lower in the melatonin treatment than in the control. The weight loss in the control reached a maximum value of 19% at 6 days, whereas the level of weight loss was only 12% in the melatonin treatment, indicating that the weight loss of baby mustard was substantially reduced by melatonin during postharvest storage.

### Chlorophyll and Carotenoids

**FIGURE 3:**
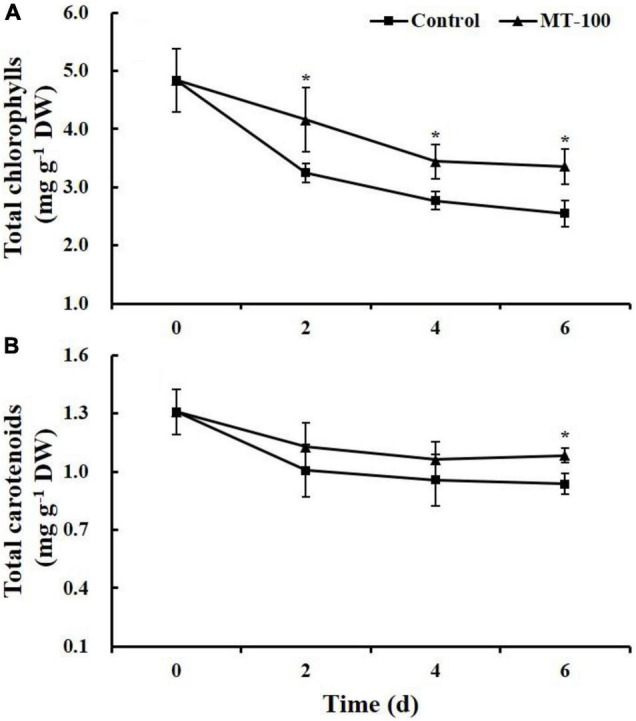
Total chlorophyll **(A)** and carotenoid **(B)** content of 100 μmol L^–1^ melatonin treated lateral buds of baby mustard during storage at 20°C. MT-100, 100 μmol L^–1^ melatonin treatment. Each value is presented as the mean ± standard error of four biological replicates. Asterisks (*) indicate the significant differences (*P* < 0.05) between control and melatonin-treated lateral buds during storage.

The total chlorophylls content in the control and melatonin-treated samples decreased during storage (Figure 3A). However, the content in the melatonin-treated group was always significantly higher than that of the control, which was consistent with the change in the acceptance score (Figure 2F). At 6 days, the total chlorophylls content in the treated samples was 1.3-fold higher compared with the control, with reductions of 31 and 47%, respectively, compared with day 0.

The total carotenoids content in the control significantly decreased over the first 2 days of storage and then remained stable. The total carotenoids content in the treated group was significantly lower at 6 days than at 0 day. However, at 6 days of storage, the total carotenoids content in samples treated with 100 μmol L^–1^ melatonin was 1.2-fold higher compared with the control (Figure 3B).

### Ascorbic Acid and Total Phenolics

**FIGURE 4:**
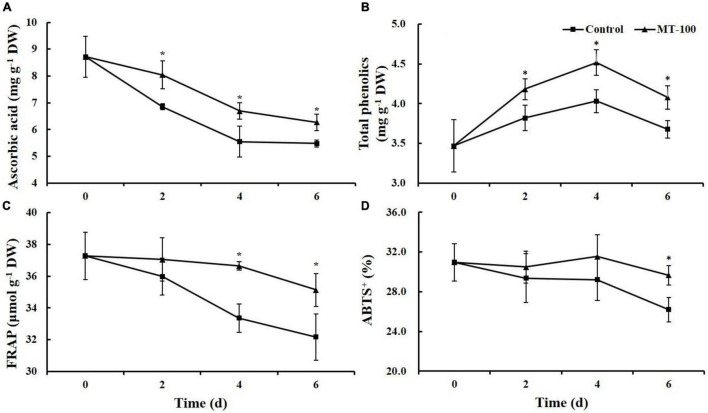
Main antioxidants content and antioxidant capacity levels of 100 μmol L^–1^ melatonin treated lateral buds of baby mustard during storage at 20°C. **(A)** Ascorbic acid; **(B)** total phenolics; **(C)** FRAP; **(D)** ABTS^+^. MT-100, 100 μmol L^–1^ melatonin treatment. Each value is presented as the mean ± standard error of four biological replicates. Asterisks (*) indicate the significant differences (*P* < 0.05) between control and melatonin-treated lateral buds during storage.

The content of ascorbic acid decreased in both the control and melatonin treatment during storage. Nevertheless, melatonin treatment significantly inhibited the decrease in the content of ascorbic acid during the entire storage period; the ascorbic acid content was 17, 21, and 14% higher than the control at 2, 4, and 6 days, respectively (Figure 4A).

The total phenolics content increased early during storage and decreased thereafter. The maximum values of the total phenolics content in both groups were observed at 4 days. During storage, the total phenolics levels in treated baby mustard were significantly higher compared with the control. The total phenolics content was 11% higher in melatonin-treated samples than in the control at 6 days (Figure 4B).

### Antioxidant Capacity

Ferric reducing antioxidant power and 2,2-azinobis (3-ethyl-benzothiazoline-6-sulfonic acid) (ABTS) levels decreased in control baby mustard during storage but were basically maintained in the melatonin treatment. The decrease in FRAP was significantly lower in melatonin-treated baby mustard than in the control at 2 days of storage. The FRAP levels were 10 and 9% higher in the melatonin treatment than in the control at 4 and 6 days, respectively (Figure 4C). The ABTS level was significantly higher (by 13%) in the melatonin-treated baby mustard than in the control at the end of storage (Figure 4D).

### Glucosinolates

**FIGURE 5:**
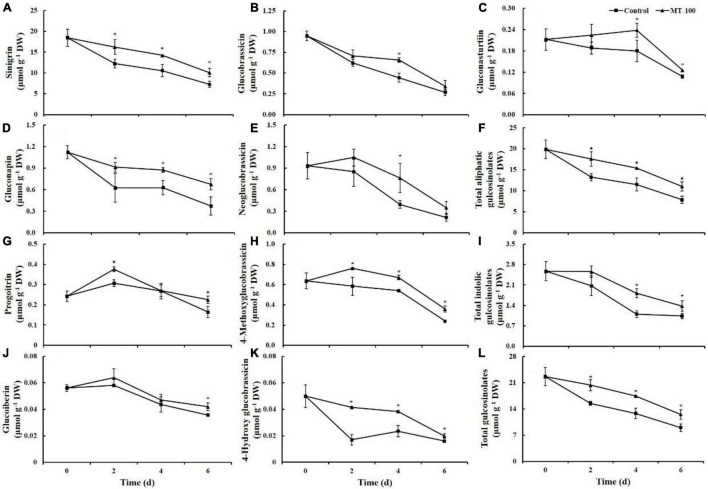
Glucosinolate content of 100 μmol L^–1^ melatonin treated lateral buds of baby mustard during storage at 20°C. **(A)** Sinigrin; **(B)** glucobrassicin; **(C)** gluconasturtiin; **(D)** gluconapin; **(E)** neoglucobrassicin; **(F)** total aliphatic gulcosinolates; **(G)** progoitrin; **(H)** 4-methoxyglucobrassicin; **(I)** total indolic gulcosinolates; **(J)** glucoiberin; **(K)** 4-hydroxy glucobrassicin; **(L)** total gulcosinolates. MT-100, 100 μmol L^–1^ melatonin treatment. Each value is presented as the mean ± standard error of four biological replicates. Asterisks (*) indicate the significant differences (*P* < 0.05) between control and melatonin-treated lateral buds during storage.

Nine glucosinolates, including four aliphatic glucosinolates, four indolic glucosinolates, and one aromatic glucosinolate, were detected in the lateral buds of baby mustard (Figure 5). Among aliphatic glucosinolates, the content of sinigrin and gluconapin in control lateral buds decreased significantly during storage by 61 and 67% at 6 days, respectively. In contrast, the content of sinigrin and gluconapin in the melatonin-treated samples decreased significantly with a loss of no more than 45% at 6 days (Figures 5A,D). The content of progoitrin and glucoiberin increased slightly in the first 2 days of storage and then decreased substantially in both the control and melatonin treatment. Nevertheless, the content of progoitrin and glucoiberin was 37 and 17% higher, respectively, in melatonin-treated samples than in the control at 6 days (Figures 5G,J). Because of the large proportion of sinigrin, the changes in the total aliphatic and total glucosinolate content were similar to the change in sinigrin: their content was 1.4-fold higher in melatonin-treated baby mustard than in the control at 6 days (Figures 5F,L).

For indolic glucosinolates, the glucobrassicin and neoglucobrassicin content in the control decreased by 58 and 53% at 4 days, respectively. The content of glucobrassicin and neoglucobrassicin was 1.9- and 1.5-fold, respectively, in the melatonin treatment compared with the control at 4 days (Figures 5B,E). The 4-methoxyglucobrassicin and 4-hydroxyglucobrassicin content in the treated samples were significantly higher compared with the control throughout storage, and no difference was observed in the total indolic glucosinolate content in the first 2 days because of the two other predominant glucosinolates (Figures 5H,K,I).

Gluconasturtiin was the only type of aromatic glucosinolate detected in baby mustard. The gluconasturtiin content was 1.3- and 1.2-fold higher in the melatonin treatment than in the control at 4 and 6 days, respectively (Figure 5C).

### Time-Related Trajectory Analysis

**FIGURE 6:**
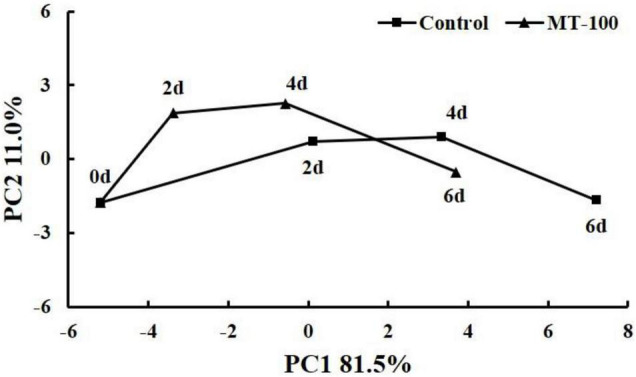
Time-related trajectory plot showing the time-related responses of sensory and nutritional qualities in the lateral buds of baby mustard plants under 100 μmol L^–1^ melatonin treatment during storage.

The different storage times and treatments of baby mustard were separated, and greater distances from day 0 corresponded to a greater degree of deterioration of postharvest baby mustard. Both control and melatonin-treated groups showed large changes in their distances in the last 2 days. The control showed the longest distance change in the first 2 days. The melatonin-treated group had shorter distance changes than the control group throughout storage. The distance from the origin under the melatonin treatment at 4 days was even shorter than that in the control at 2 days and approximately half of that in the control at 6 days (Figure 6). These results indicate that the postharvest deterioration of baby mustard was clearly delayed by the 100 μmol L^–1^ melatonin treatment.

## Discussion

The ability of melatonin to extend the shelf life and improve the quality of postharvest fruits and vegetables has been previously studied, and the optimal concentration for postharvest preservation differs among plant species (Zhang et al., 2020; Wu X. et al., 2021). For example, 1,000 μmol L^–1^ melatonin treatment was optimal for delaying the lignification of bamboo shoots, as this treatment significantly decreased the degree of firmness, as well as the lignin and cellulose content (Li et al., 2019). Low doses of melatonin (1 μmol L^–1^) were optimal for preserving antioxidants in broccoli florets, such as ascorbic acid, carotenoids, and total phenolics, as well as glucosinolates (Miao et al., 2020). In our study, treatment with 100 μmol L^–1^ melatonin was more effective for extending the shelf life of baby mustard (Figure 1), which is consistent with Chinese flowering cabbage and several types of fruits, such as peach (Cao et al., 2016; Gao et al., 2016), pear (Zhai et al., 2018; Zheng et al., 2019), sweet cherry (Wang F. et al., 2019; Miranda et al., 2020), and pomegranate (Jannatizadeh, 2019). In addition to plant species, the optimal concentrations of melatonin can vary with the stages of fruit development, different treatment conditions (e.g., application method and duration), and storage conditions (Ma et al., 2016; Onik et al., 2021).

Melatonin treatment improves the sensory quality of vegetables. In this study, melatonin-treated baby mustard had higher sensory parameter scores than control baby mustard (Figure 2B-F). The color and acceptance scores of baby mustard were correlated with the chlorophyll content and yellowing. Melatonin treatment markedly inhibited the chlorophyll degradation of baby mustard (Figure 3A). The protective effect of melatonin on chlorophyll has been observed in previous studies of barley leaves (Arnao and Hernández-Ruiz, 2009) and detached apple leaves (Wang et al., 2012). Tan et al. (2019) found that melatonin treatment suppressed the expression of chlorophyll catabolic and senescence marker genes in Chinese flowering cabbage. Similar results have been obtained with broccoli florets (Miao et al., 2020; Wu C. H. et al., 2021). Melatonin treatment can keep the chloroplasts intact as well as inhibit the expression of genes and the activity of enzymes involved in chlorophyll degradation (Wu C. H. et al., 2021). The results of our study indicated that melatonin can prevent chlorophyll from degrading. Fruits and vegetables are highly susceptible to water loss because of their metabolic activity, respiration, and transpiration after harvest (Bal, 2019). The 100 μmol L^–1^ melatonin treatment significantly decreased the weight loss of baby mustard throughout the storage period compared with the control. This may be ascribed to the increased skin strength under melatonin treatment (Liu et al., 2018; Miranda et al., 2020). Melatonin treatment has also been shown to significantly reduce the weight loss of peach (Gao et al., 2016) and strawberry (Liu et al., 2018) fruits during storage.

Antioxidants, such as carotenoids, ascorbic acid, and phenolics, are important phytochemicals in baby mustard that protect cells against oxidative stress (Xiao et al., 2012; Sun et al., 2018; Moloto et al., 2020). The content of carotenoids and ascorbic acid decreased during postharvest storage period, and melatonin treatment reduced the magnitude of the decrease (Figures 3B, 4A). The content of total phenolics increased continuously during the first 4 days of storage and decreased thereafter (Figure 4B), which could be caused by postharvest stress; these findings are consistent with the results of Miao et al. (2020) showing that phenolic compounds increased in broccoli florets at 5 days of storage at 20°C. Melatonin exposure delayed the decrease in FRAP and ABTS^+^ levels in postharvest baby mustard. Similar results have also been obtained in other studies of strawberry (Liu et al., 2018), plum (Bal, 2019), and pomegranate (Aghdam et al., 2020). Melatonin plays a role not only as an antioxidant by scavenging ROS directly but also augment the activity of antioxidant enzymes and the content of other antioxidants mentioned above and thus increases the postharvest life of fruits and vegetables (Ahammed et al., 2020). Conferring delaying the senescence in sweet cherries (Wang F. et al., 2019) and chilling tolerance in pomegranate (Jannatizadeh, 2019) were partly ascribed to the higher SOD, CAT, and APX activity under the melatonin treatment, which attenuated H_2_O_2_ accumulation. Moreover, melatonin treatment indued the expression of the key gene *PAL*, which encodes an enzyme in the phenylpropanoid pathway, contributing to the accumulation of total phenolics in postharvest fresh-cut pear (Zheng et al., 2019). These findings differ slightly from those obtained for bamboo shoots (Li et al., 2019). Melatonin treatment increased the antioxidant potential of bamboo shoots during postharvest storage; however, it significantly inhibited PAL activity compared with control shoots. PAL might also play a role in catalyzing the polymerization of monolignols in the lignification of bamboo shoots, and the effect of melatonin on PAL activity varies among species and organs (Boudet, 2000; Mastropasqua et al., 2016).

Glucosinolates are important health-promoting compounds unique to *Brassica* vegetables (Jia et al., 2009; Li et al., 2018; Wang J. S. et al., 2019; Wu et al., 2019; Zeng et al., 2021). Thus, maintaining the content of glucosinolates in postharvest baby mustard is essential for maintaining its nutritional quality. Our results indicated that individual and total glucosinolates content in postharvest baby mustard decreased during storage at room temperature, which was accompanied by a high rate of senescence (Figure 5). However, the decrease was alleviated by melatonin treatment; the total glucosinolate content was 1.4-fold higher in melatonin-treated baby mustard than in control baby mustard at the end of storage (Figure 5L). As baby mustard aged, cellular integrity was destroyed, and vacuoles burst, which made glucosinolates and myrosinase come into contact, resulting in the degradation of glucosinolates (Sun et al., 2012). However, the contact between glucosinolates and myrosinase was delayed under melatonin treatment because cell structures were preserved, which helped maintain the glucosinolate content (Arnao and Hernández-Ruiz, 2018). Melatonin treatment has also been shown to be effective in preserving glucosinolates in postharvest broccoli (Miao et al., 2020; Wei et al., 2020).

Safety is a critical point for the consumer. Melatonin is approved as a dietary supplexment by the United States Food and Drug Administration (FDA), and can be purchased over-the-counter in both the United States and Canada (Foster, 2021). Ministry of Public Health of China has also approved melatonin as health products (Liu and Sun, 2017). There’s not enough information yet about possible side effects but evidence suggests that melatonin supplements promote sleep and are safe for short-term use (National Center for Complementary and Integrative Health, 2021). The concentration of melatonin used as a postharvest treatment on horticultural crops ranges from 1 to 1,000 μmol L^–1^, and generally adopts the application method of immersing or spraying. In our experiment, the optimal concentration of the melatonin solution for immersing is 100 μmol L^–1^, and the residual melatonin in lateral bud is very limited compared with the usual dose of melatonin (1–3 mg per day) as a dietary supplement (Lutterschmidt et al., 2003). Morevoer, as for people, melatonin is not easy to accumulate in the body: the half-life is short; and there is a liver metabolic pathway, 70% of the metabolites are excreted in the urine (Lutterschmidt et al., 2003; Bagci et al., 2011). Even if consumers still have concerns, the light-decomposing properties of melatonin can reassure them: exposure to light after melatonin treatment can reduce the residue (Ma et al., 2021). Therefore, melatonin treatment is safe to use for postharvest preservation of fruit and vegetables.

In sum, application of 100 μmol L^–1^ melatonin was the most effective for prolonging the shelf life and maintaining the quality of postharvest baby mustard. Treatment with 100 μmol L^–1^ melatonin delayed sensory quality deterioration by inhibiting the degradation of chlorophyll and decreasing the degree of yellowness; sustained the antioxidant potential of postharvest baby mustard by maintaining higher levels of antioxidants and antioxidant capacity; and slowed decreases in the content of individual and total glucosinolates. Treatment with 100 μmol L^–1^ melatonin is effective for preserving the postharvest quality of baby mustard.

## Data Availability Statement

The original contributions presented in the study are included in the article/supplementary material, further inquiries can be directed to the corresponding authors.

## Author Contributions

HD and YW: investigation and writing—original draft preparation. ZL: data curation and writing—original draft preparation. YiZ, JB, JX, YaZ, and RG: data curation. HL: investigation and funding acquisition. FZ: conceptualization, funding acquisition, and writing—reviewing and editing. BS: funding acquisition, writing—reviewing and editing, and conceptualization. All authors: review and editing.

## Conflict of Interest

The authors declare that the research was conducted in the absence of any commercial or financial relationships that could be construed as a potential conflict of interest.

## Publisher’s Note

All claims expressed in this article are solely those of the authors and do not necessarily represent those of their affiliated organizations, or those of the publisher, the editors and the reviewers. Any product that may be evaluated in this article, or claim that may be made by its manufacturer, is not guaranteed or endorsed by the publisher.
